# The Effect of Thermal Annealing on the Structure and Gas Transport Properties of Poly(1-Trimethylsilyl-1-Propyne) Films with the Addition of Phenolic Antioxidants

**DOI:** 10.3390/polym15020286

**Published:** 2023-01-05

**Authors:** Anton Kossov, Vladimir Makrushin, Ivan Levin, Samira Matson

**Affiliations:** A.V. Topchiev Institute of Petrochemical Synthesis, Russian Academy of Sciences, Leninsky Prospect 29, 119991 Moscow, Russia

**Keywords:** poly(trimethylsilyl-1-propyne), permeability, polymer annealing

## Abstract

The thermally activated relaxation of poly(1-trimethylsilyl-1-propyne) (PTMSP) samples of various *cis*-/*trans*-compositions (50–80% units of *cis*-configuration) in the presence of phenolic antioxidants of various structures was investigated. It was pointed out that polymers with a high content of *cis*-units exhibited greater thermal-oxidative stability due to the greater flexibility of the *cis*-enriched macrochains. The use of hindered phenols as antioxidants made it possible to prevent the process of thermally initiated oxidative degradation. At the same time, the most effective stabilizing agents were antioxidants with larger molecules such as Vulkanox BKF, Irganox 1010, and Irganox 1076. It was shown that the permeability coefficients of stabilized PTMSP during thermal treatment initially slightly decreased (by 20–30%), which, according to the X-ray diffraction data, was associated with an increase in the density of the macrochain packing, and during further heating remained practically unchanged. Note that for the *cis*-enriched samples, no signs of oxidation or decrease in the transport characteristics were observed during polymer heating for 240 h at 140 °C.

## 1. Introduction

The active development of membrane separation technologies is due to a number of advantages such as the relative ease of operation and lower cost compared to other separation technologies such as distillation. The evaluation and study of gas transport properties for a variety of polymers have been carried out. However, only approximately ten out of several hundred polymers have found commercial use [1]. One of the main reasons for this is physical aging, which leads to a significant decrease in the membrane characteristics of the polymer [2]. Physical aging is typical to some extent for all glassy polymers. At the same time, in polymers with a high free volume, physical aging proceeds faster.

Such polymers include glassy 1,2-disubstituted polyacetylene poly(1-trimethylsilyl-1-propyne) (PTMSP). This polymer has a uniquely high fraction of free volume (approximately 0.3) and a microporous organization, which leads to an extremely high permeability for gases and vapors, as well as the selectivity of the release of organic vapors from mixtures with noncondensable gases [3,4,5,6,7,8]. However, for PTMSP, there is a noticeable decrease in the proportion of free volume over time and, as a result, a drop in the permeability coefficients due to the relaxation of macrochains [9,10,11,12,13].

Thermal annealing is one of the simple and effective methods to regulate the organization and porous structure of a polymer membrane. During heat treatment, the relaxation of polymer segments can be controlled by changing the temperature, which contributes to the rapid achievement of thermodynamic equilibrium. Controlled thermal annealing also makes it possible to obtain membranes with increased selectivity for various gas separation regions [14,15].

The thermal treatment of PTMSP membranes, as shown in Refs. [16,17], leads to an increase in the ideal selectivity with respect to a number of gases, including noncondensable ones such as hydrogen. Thus, the thermal annealing of PTMSP can be an effective way to achieve both the stability and selectivity of this high-free-volume glassy polymer.

PTMSP is fairly thermally stable due to the screening of double bonds of the main chain by bulky substituents [18]. Nevertheless, heating in oxygen-containing media can lead to its destruction due to oxidation. For most synthetic polymers, a radical mechanism of oxidative degradation has been proposed, the active particle of which is the peroxyl radical ROO· [19,20]. To solve the problem of thermal-oxidative degradation, small additives of antioxidants can be used to inhibit the degradation process, which makes it possible to prolong the service life of the polymer material [20,21,22].

According to the mechanism of action, antioxidants of the first and second types are distinguished [21]. The former act as donors of hydrogen atoms, which terminate free radicals. Sterically hindered phenols and aromatic amines are commonly used as stabilizers of this type. Phenols are more commonly used since they are less toxic and do not affect the color of the polymer material, unlike amines [22]. Among the antioxidants of this group, Irganox 1010 (pentaerythritol tetraoxy(3-(3,5-ditertbutyl-4-hydroxyphenyl)propionate), Irganox 1076 (octadecyl-3-(3,5-ditertbutyl-4-hydroxyphenyl)-propionate), and Irganox HP-136 (5,7-ditert-butyl-3-(3,4-dimethylphenyl)3Н-benzofuran-2-one are most frequently used [23].

The effect of the introduction of antioxidants into the polymer matrix of such rigid-chain loosely packed polymers as PTMSP may result in the protection against thermal-oxidative degradation, i.e., the chemical aging of the polymer. Antioxidants can also prevent the physical aging of this class of polymers. The incorporation of bulky antioxidant molecules into the polymer matrix makes it possible to avoid the strong compaction of macrochains and, as a consequence, decreases the fraction of free volume as a result of polymer relaxation over time.

The main problem that arises when using antioxidants is associated with the low compatibility of many low-molecular-weight components with the polymer material, which ultimately leads to the migration of the stabilizing agent inside the polymer matrix due to diffusion processes and leaching from the polymer upon contact of the material with various aqueous or organic media. To prevent these undesirable processes, various approaches have been proposed, for example, the use of high-molecular-weight components of a branched structure (dendrimers) containing fragments of sterically hindered phenol-type antioxidants [21], the copolymerization with vinyl derivatives of hindered phenols [24,25], and the introduction of nanoparticles containing phenolic groups into the polymer [26], as well as the functionalization of the polymer by introducing functional groups of antioxidants covalently bonded to the surface of the material [27]. Examples of the grafting of phenol-type antioxidants onto the surface of various linear polymers, including chitosan [28], polybutadiene [29], polystyrene [30], polyethylene [31,32], polypropylene [33,34], and polyisobutylene [35], have been described. In all cases, there was a significant increase in the temperature of the onset of thermal-oxidative degradation of the material compared to the unmodified polymer.

**Figure 1:**
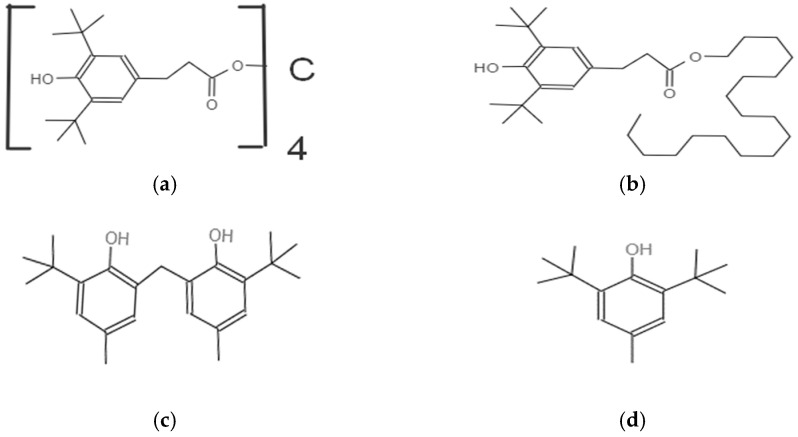
Structures of the antioxidants: (**a**) Irganox 1010 (penthaeritrytetetraoxy(3-(3,5-ditert-buthyl-4-hydroxyphenyl)propyonate); (**b**) Irganox 1076 (octadecyl-3-(3,5-ditert-buthyl-4-hydroxyphenyl)-propyonate); (**c**) Vulkanox BKF (2,2-methylene-bis(4-methyl-6-tert-buthylphenol); (**d**) Vulkanox BHT (4-methyl-2,6-ditert-buthylphenol).

In general, published data indicate that the compatibility of an antioxidant with a polymer material, as can be judged from the mechanical properties, the yellowness index of the polymer containing the antioxidant compared to the original, and the resistance of the material to solvent extraction, generally increases with increasing molecular weight and degree of branching of the structure of the antioxidant. In this work, we chose a number of phenol-type antioxidants of different structure of the benzene ring substituents to study their effect on the thermal-oxidative stability and stability of the gas transport characteristics of PTMSP during thermal annealing. The structural formulas of the antioxidants used are shown in Figure 1.

## 2. Materials and Methods

### 2.1. Initial Compounds: Synthesis and Purification

The monomer 1-trimethylsilyl-1-propyne was obtained using the reaction of methylacetylene from the methylacetylene-allene fraction with an alkylmagnesium halide followed by treatment of the reaction mixture with trimethylchlorosilane [36]. The monomer purity of 99.9% was controlled using clear distillation.

The solvents cyclohexane (99.8%, Fisher Scientific, Waltham, MA, USA) and toluene (99.97%, Fisher Scientific, Waltham, MA, USA) were distilled three times with CaH_2_ in high-purity argon before polymerization.

Carbon tetrachloride was purified with a 10% sodium hydroxide aqueous solution and washed three times with water. Next, it was dried with CaCl_2_ for 48 h, after which it was distilled three times with P_2_O_5_ in a high-purity argon atmosphere.

Niobium pentachloride NbCl_5_ (99.9%, Fluka Chemie AG, Buchs, Switzerland), tantalum pentachloride TaCl5 (99.9%, Fluka Chemie AG, Buchsm, Switzerland), niobium pentabromide NbBr_5_ (99.9%, ABCR, Karlsruhe, Germany), and Ph_4_Sn cocatalyst (>98.0%, TCI Ltd., Tokyo, Japan) were used without additional cleaning.

PTMSP was synthesized according to the procedure described in Ref. [37].

Antioxidants Vulkanox BKF (2,2-methylene-bis(4-methyl-6-tert-butylphenol), Vulkanox BHT (4-methyl-2,6-ditert-butylphenol), and Irganox 1010 (pentaerythritol tetraoxy(3-(3,5-ditretbutyl- 4-hydroxyphenyl)propionate) (nortex) were used as received.

The intrinsic viscosity values of the PTMSP samples were measured in CCl_4_ at 25 °С in an Ostwald–Ubbelohde viscometer.

The molecular weights of the polymers were determined using GPC of the polymer solutions in cyclohexane using a Waters 600 Powerline system equipped with two mixed-C Pl-gel columns (Polymer Laboratories) and detectors—a Waters 410 differential refractometer and a light scattering Wyatt Dawn (cyclohexane solvent, T = 60 °C, flow rate 1 mL/min).

The ratio of *cis*-/*trans*-units in the PTMSP samples was determined from the ^13^C NMR spectra of the polymer solutions in C_6_D_12_. The spectra were recorded on an Avance series spectrometer (Bruker BioSpin GmbH, Ettlingen, Germany) in the single-pulse mode with broadband decoupling from protons during the free induction decay signal and a reduced decoupling power during the relaxation delay (duration 3 s), which made it possible to preserve the signal amplification due to the Overhauser effect. The spectral capture width was 250 ppm. The accumulation time of the free induction decay signal was 12–18 h. The spectra were analyzed using the ACD/Labs program (Advanced Chemistry Development, Inc., Toronto, ON, Canada, version 12.0 for Microsoft Windows). The quantitative ratio of *cis*- and *trans*-units in the polymers was calculated according to the method we developed earlier and described in Ref. [37]. The calculation was performed using the ratio of the intensities of the peaks of the doublet signals with chemical shifts 152.0–152.8 ppm (*cis*-configuration) and 151.0–151.3 ppm (*trans*-configuration) corresponding to the carbon atom =C-CH_3_, and with chemical shifts 139.6–140.0 ppm (*cis*-configuration) and 137.7–138.5 ppm (*trans*-configuration) corresponding to the carbon atom =C-Si.

### 2.2. Preparation of PTMSP Films

The films of the polymers were constructed by pouring the polymer solutions in cyclohexane (1.5% wt.) onto cellophane, drying in air at 20 °C for 7 days, and then drying under vacuum for 48 h. The film thickness varied from 30 to 50 μm.

The stabilized PTMSP films were obtained using a similar procedure from polymer solutions in cyclohexane, in which 2% wt. antioxidant was dissolved.

### 2.3. Heat Treatment of PTMSP Films

The thermal annealing of the polymer films was carried out in a BINDER FD 53 oven (Binder GmbH, Tuttlingen, Germany) equipped with an electronic controller with a LED digital display in air. The temperature was displayed with an accuracy of one degree. The cabinet was electrically heated and ventilated with forced circulation by means of a fan. The inner chamber, the preheating chamber, and the inner side of the doors were composed of stainless steel (material no. 1.4301). The films in the Petri dishes were placed in a dry oven, after which the heating of the chamber began. The set temperature (120–150 °С) was reached within 15–20 min. After a predetermined heating time, the films were cooled in an oven for 3 h.

### 2.4. X-ray Diffraction Study

The diffraction patterns of the polymer samples were obtained using a Rigaku Rotaflex RU-200 X-ray diffractometer (Rigaku Co., Ltd., Tokyo, Japan) with a rotating copper anode (characteristic radiation wavelength 0.1542 nm). The flat films were fixed in 6 layers on an aluminum frame, and the shooting was carried out in the “transmission” geometry in the angular range of 2.5–50 degrees in 2θ according to the Bragg–Brentano scheme. Next, the resulting diffraction patterns were processed using the Fityk program; after subtracting the background line, they were presented as a sum of several Gauss peaks. The positions of these peaks were recalculated into the interplanar spacing using the Wulf–Bragg formula.

### 2.5. Thermogravimetric Analysis (TGA)

A TGA was carried out in air on a “Mettler Toledo TGA/DSC-3+” instrument (Mettler Toledo, Greifensee, Switzerland) in the temperature range 20–1000 °С under the flow rate of air of 50 mL/min. The weighed samples (5–40 mg) were placed in aluminum oxide crucibles with a volume of 70 μL. The heating rate was 10 °C/min. The measurement error for determining the temperature was 0.3 °C, and for determining the mass was 0.1 µg.

### 2.6. Gas Permeability Measurements

The parameters of the film permeability of the polymer samples were determined at 25 °С using a setup operating on the “constant volume/variable pressure” principle. The work was based on the manometric method for measuring the flow rate of gas passing through the membrane. The flow rate of the gas that had passed through the membrane was determined using the time of its leakage into the calibrated evacuated volume. The membrane permeability coefficients were calculated using the formula:

P (barrer) = 10^10^ × V × Δр × h/(t × S × p_in_ × 760 × 76), (1)

where V is the calibrated pumped volume (1175 mL); S is the membrane area (24.18 cm^2^); h is the membrane thickness (cm); p_in_ is the pressure drop across the membrane (up to 10 atm); t is the time of gas leakage into the volume V from pressure p1 to pressure p2 (the difference Δр (tor) = p2 − p1 can be chosen from the series 1, 2, 5, 10, or 20 torr), which determine the moments of turning on and off the stopwatch.

## 3. Results

### 3.1. Gas Transport Properties of PTMSP Films

**Figure 2:**
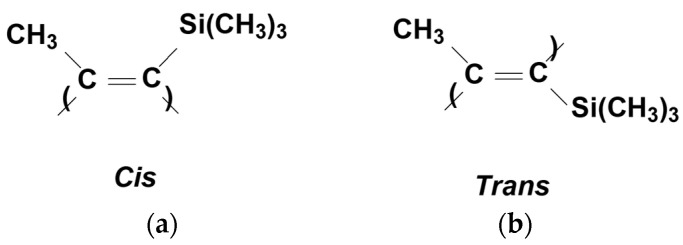
Repeating units of PTMSP with (**a**) *cis*- and (**b**) *trans*-configuration.

Alternating double bonds in PTMSP macromolecules can have both *cis*- and *trans*-configurations (Figure 2). Quantitative ratios of *cis*- and *trans*-units in polymers depend on the synthesis conditions, and as it was shown in earlier works [37,38], the geometric structure (*cis*-/*trans*-ratio) of PTMSP determines its morphology, which controls the dissolution ability as well as the membrane characteristics. Therefore, to study the effect of stabilizers on the stability of the gas transport properties of PTMSP, we chose three samples of different *cis*-/*trans*-compositions. Table 1 lists the synthesis conditions and characteristics of the PTMSP samples selected to study the effect of phenolic stabilizers of various chemical structures on the thermal-oxidative stability and transport characteristics of isotropic films. The content of *cis*-units in the samples ranged from 50 to 80%.

Table 2 shows the values of the permeability coefficients and the ideal selectivity for freshly prepared isotropic films of pure PTMSP and PTMSP containing stabilizers Irganox 1010, Vulkanox BKF, and Vulkanox BHT. For comparison, data are also provided for the antioxidant Irganox 1076, the effectiveness of which was demonstrated in our earlier works [39].

It can be seen that the addition of stabilizers led to a decrease in the permeability of the individual gases compared to the pure polymer films. The greatest drops in the gas permeability coefficients were observed in films prepared using the Vulkanox BKF stabilizer (up to 60%). The introduction of Irganox 1010 and Vulkanox BHT stabilizers led to a much smaller permeability decrease (no more than 30%). The decrease in permeability, apparently, is a consequence of the longer trajectory of the penetrant molecule through the polymer matrix when the stabilizer is introduced. On the other hand, the degree of the drop in permeability depending on the stabilizer is determined both by the size and the structure of the stabilizer–antioxidant molecule and by the size of the free-volume elements in the polymer matrix, in which the stabilizer molecules can be located. Thus, the greater relative decrease in the permeability of PTMSP with C2 compared to PTMSP with C3 could be caused by the longer winding trajectory of gas molecules due to the larger volume of the Vulkanox BKF antioxidant molecules. At the same time, the samples of PTMSP with C1 and PTMSP with C3 had almost the same level of permeability, which can be explained by the correspondence between the sizes of the extremely bulky Irganox 1010 stabilizer molecules and the free-volume elements of PTMSP occupied by these molecules. At the same time, smaller pores remained open for the transfer of gas molecules through the polymer matrix.

### 3.2. Thermal Annealing of PTMSP Films

**Figure 3:**
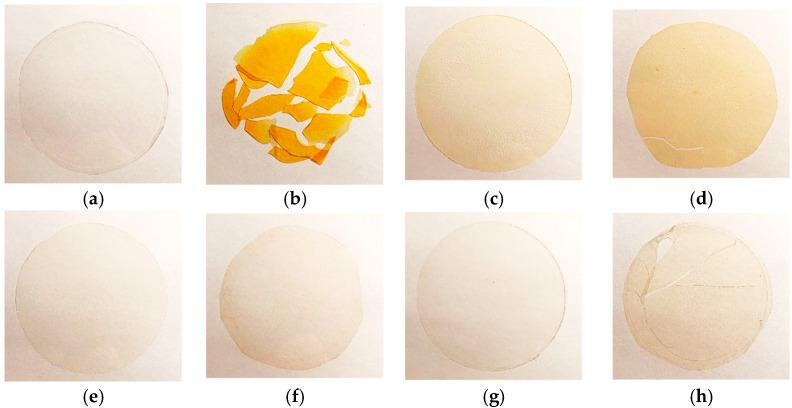
Visual appearance of pure and stabilized PTMSP films before and after heat treatment (140 °C): pure PTMSP-3 before (**a**) and upon 24 h of heat treatment (**b**); PTMSP-1 with Irganox 1010 before (**c**) and upon 72 h of heat treatment (**d**); PTMSP-3 with Irganox 1010 before (**e**) and upon 240 h of heat treatment (**f**); PTMSP-3 with Vulkanox BHT before (**g**) and upon 150 h of heat treatment (**h**).

To study the effect of sterically hindered phenolic stabilizers of various structures on the stability of PTMSP films, the samples were heated at 140 °C. The results of the permeability measurements of the stabilized films subjected to heat treatment are presented in Table 3, Table 4 and Table 5. The PTMSP films containing no stabilizers after heating at 140 °C for 24 h were destroyed, which was probably due to the partial oxidation of the polymer. For example, Figure 3a,b presents the visual changes of the pure PTMSP-3 film before and upon heat treatment (at 140 °C, duration 24 h). With the introduction of stabilizers, the films of the PTMSP samples became more resistant to heating, but to a different extent. Thus, the permeability coefficients of the PTMSP-1 films of a mixed *cis-*/*trans*-composition with the addition of any of the three stabilizers increased after heating at 140 °C for 48 h, and after heating for 72 h the films lost their mechanical properties (became brittle) (Figure 3c,d). The PTMSP-2 and PTMSP-3 films enriched with *cis*-units with all stabilizers exhibited a significantly higher thermal and oxidative stability. When stabilizers Irganox 1010 and Vulkanox BKF were added, the PTMSP-2 films remained stable after total heating for at least 240 h. As an example, there was only a slight change in the color of the PTMSP-3 film with added Irganox 1010 after heat treatment during the 240 h, and the film sample retained its flexibility and strength (Figure 3e,f). The Vulkanox BHT stabilizer showed the lowest efficiency. The permeability of the PTMSP-2 and PTMSP-3 films increased after heating for 100 h, which indicated irreversible changes in the polymer structure associated with its degradation. Further heating led to brittleness and the destruction of the sample (Figure 3g,h).

The data show that in a series of polymers containing from 50 to 80% *cis*-units, PTMSP-1 with a mixed microstructure had the lowest thermal-oxidative stability. The stabilized samples PTMSP-2 and PTMSP-3 with an increased content of *cis*-units, 70 and 80%, respectively, exhibited a significantly higher resistance to thermal-oxidative degradation.

In earlier works [38], it was shown that PTMSP macromolecules enriched with *cis*-units exhibit greater thermodynamic flexibility than chains of mixed *cis*-/*trans*-composition, as evidenced by the different values of the Kuhn segment of these polymers: ~3.3 × 10^−7^ cm (13–14 units) and ~7 × 10^−7^ cm (36 units) for *cis*- and *trans*-enriched samples, respectively. Macromolecules with a higher content of *cis*-units are likely to take a more folded conformation, in which the main chain is shielded by bulky Si(CH_3_)_3_ substituents to a greater extent than in polymers with a higher content of *trans*-units, which makes it less accessible for the attack of the peroxyl radicals arising during the oxidation of the polymer, and, accordingly, leads to a greater thermal-oxidative stability of the samples. At the same time, when Vulkanox BHT was used as a stabilizing agent, the film collapsed after 100 h of annealing at 140 °C. The addition of Irganox 1010 and Vulkanox BKF stabilizers increased the thermal stability of the PTMSP-2 and PTMSP-3 films; in both cases, no destruction of the films was observed up to 250 h of annealing at 140 °C.

**Figure 4:**
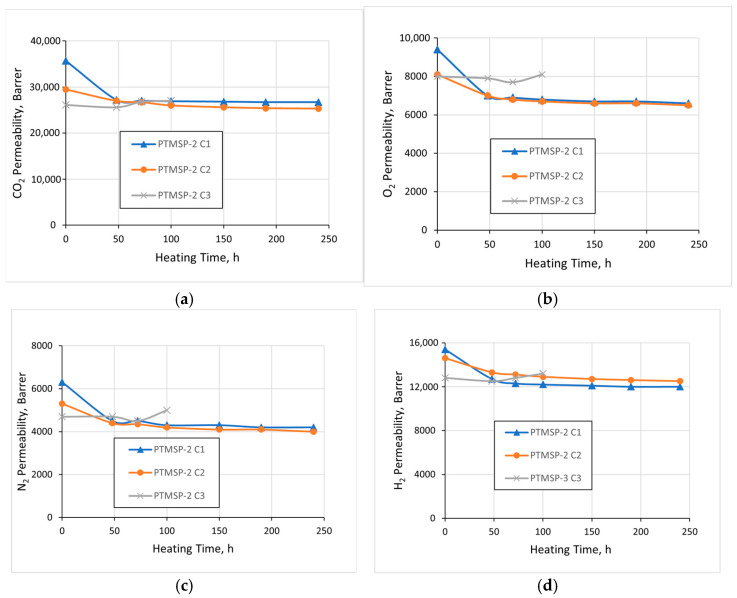
Gas permeability coefficients vs. heating time for the films of PTMSP-2 containing Irganox 1010 (С1), Vulkanox BKF (С2), and Vulkanox BHT (С3). (**a**) СО_2_; (**b**) О_2_; (**c**) N_2_; (**d**) H_2_.

**Figure 5:**
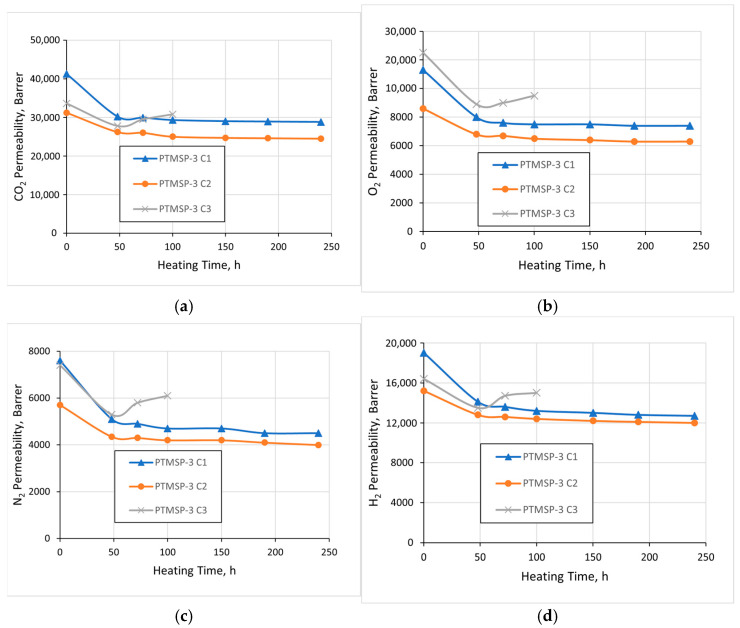
Gas permeability coefficients vs. heating time for the films of PTMSP-3 containing Irganox 1010 (С1), Vulkanox BKF (С2), and Vulkanox BHT (С3). (**a**) СО_2_; (**b**) О_2_; (**c**) N_2_; (**d**) H_2_.

Figure 4 and Figure 5 show the dependences of individual gas permeability coefficients of stabilized *cis*-enriched PTMSP-2 and PTMSP-3 samples on the heat treatment time (140 °C). It can be seen that in all cases, during the first 50 h, there was a slight drop in the permeability coefficients (by 5–20%). In the case of films stabilized with Irganox 1010 and Vulkanox BKF, a quasi-equilibrium state was achieved, in which the permeability level remained practically unchanged during further heating, and the polymer did not show any signs of thermal-oxidative degradation, at least during the 240 h of the total heating time. In the case of films with added Vulkanox BHT, the permeability of both PTMSP-2 and PTMSP-3 films noticeably increased after 100 h of heat treatment, which indicated the appearance of nonselective defects in the films’ structures due to polymer degradation. Further heat treatment led to brittleness and the destruction of the polymer films.

**Figure 6:**
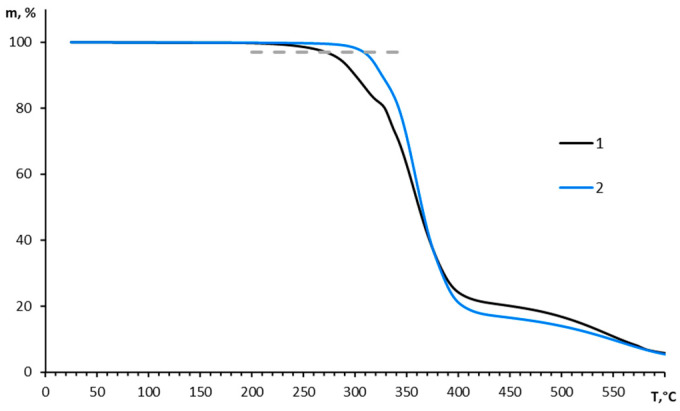
TGA curves for films with pure PTMSP-3 (1) and with Irganox 1010 (2).

Thus, the antioxidants Irganox 1010 and Vulkanox BKF proved to be the most effective inhibitors of the thermal-oxidative degradation of PTMSP. The higher thermal-oxidative stability of PTMSP with the addition of these antioxidants was also supported by TGA. As one can see in Figure 6, pure PTMSP-3 started to decompose (3% of mass loss) in air at 278 °C, while PTMSP-3 with Irganox 1010 started to decompose in air at 315 °C.

**Figure 7:**
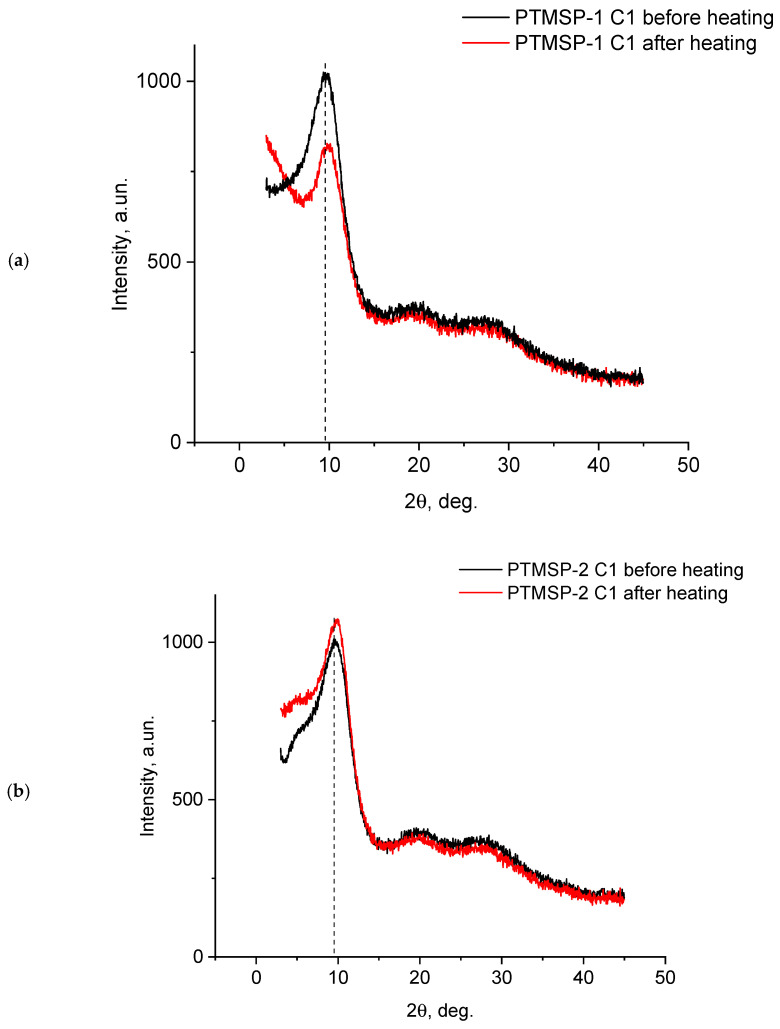

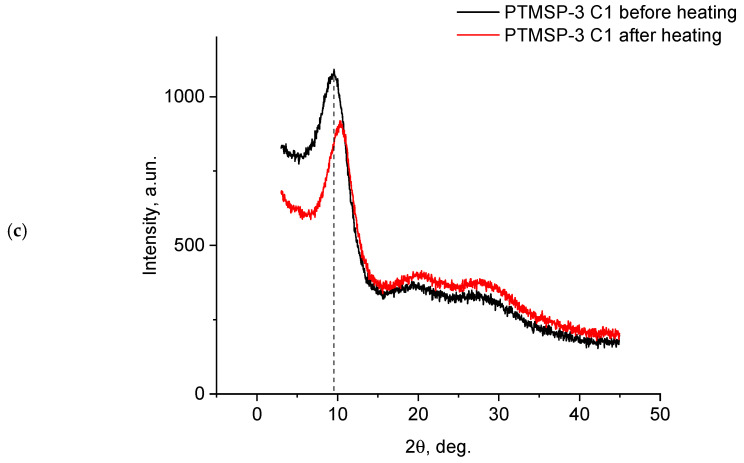
X-ray curves for the PTMSP samples containing stabilizer Irganox 1010, before and after heat treatment (140 °С, 50 h). (**a**) PTMSP-1; (**b**) PTMSP-2; (**c**) PTMSP-3.

The initial drop in film permeability during heating is caused by a decrease in the fraction of free volume due to the thermally initiated relaxation of polymer chains. This was confirmed with the X-ray analysis of the films. Figure 7 shows the X-ray diffraction patterns of the PTMSP samples with different microstructures (with Irganox 1010) before and after heating. All diffractograms clearly show a rather narrow diffraction maximum with an angular position of approximately 10 degrees, which we believed corresponded to the interchain distances in the polymer, which was calculated according to the Bragg’s equation. In all cases, there was a shift of the main maximum after heating towards larger angles (therefore there were smaller interchain distances); for the first two cases this is not as noticeable as for the third.

The X-ray diffraction data indicate a decrease in the interchain distance in the polymer matrix during heat treatment and an increase in the packing density of the PTMSP macromolecules during the thermally activated relaxation. A similar effect was noted earlier during the annealing of PTMSP films at a temperature of 120 °C [40]. An increase in the density of the polymer of ~10% was noted with a simultaneous decrease in the permeability of more than an order of magnitude during the heat treatment for 8 h. It should be noted that, in our case, the annealing of the stabilized PTMSP even at a higher temperature (140 °C) led only to a slight decrease in the permeability, while maintaining a sufficiently high level (~10^3^ Barrer).

## 4. Conclusions

As established in this work, thermal annealing makes it possible to quickly reach an equilibrium state in which the permeability coefficients practically do not change over time, while remaining at a fairly high level (10^3^–10^4^ Barrer). The use of small additives (2 wt %) of phenolic antioxidants makes it possible to avoid the process of the thermal-oxidative degradation of PTMSP. Antioxidants with the largest molecules showed the greatest efficiency: Irganox 1010, Irganox 1076, and Vulkanox BKF. Whereas the less sterically hindered phenol Vulkanox BHT appeared to be an ineffective inhibitor of PTMSP oxidation. At the same time, polymers enriched with *cis*-configuration units showed the highest thermal-oxidative stability, which was associated with their ability to adopt a more folded conformation, in which the polymer chain was shielded to a greater extent by side substituents. Thus, from the considered group of antioxidants, the derivatives of (3,5-ditretbutyl-4-hydroxyphenyl)-propionic acid, Irganox 1010, and Irganox 1076 seem to be the most preferable for PTMSP in terms of their efficiency and effect on gas transport characteristics. This allows considering thermal annealing in the presence of these phenolic antioxidants a promising way to stabilize the membrane characteristics of PTMSP for its use in real gas separation processes.

## Figures and Tables

**Table 1:** Synthesis conditions and characteristics of PTMSP.

Sample	Catalytic System ^1^	Yield, %	[η], dl/g	M_w_, 10^−3^	M_w_/M_n_	% *Cis*-units
PTMSP-1	NbCl_5_	95	0.9	280	1.2	50
PTMSP-2	NbBr_5_/Et_3_SiH	85	3.4	500	1.5	70
PTMSP-3	NbBr_5_/Ph_4_Sn	70	3.0	540	1.4	80

^1^ [M]_0_ = 1 M, [M]_0_/[Cat] = 50, [Cat] = [Cokat]. Polymerization time for samples PTMSP-2 and PTMSP-3: 5 days, Т = 80 °С, solvent: cyclohexane. Polymerization time for sample PTMSP-1: 24 h, Т = 25 °С, solvent: toluene.

**Table 2:** Individual gas permeability coefficients of freshly prepared nonstabilized PTMSP films and films containing stabilizers Irganox 1010 (С1), Vulkanox BKF (С2), Vulkanox BHT (С3), and Irganox 1076 (C4).

Sample	Permeability, P (Barrer *)				Ideal Selectivity, α	
	O_2_	N_2_	H_2_	CO_2_	O_2_/N_2_	CO_2_/N_2_
PTMSP-1	9200	5800	17,000	34,200	1.6	5.2
PTMSP-1 С1	6600	4000	13,000	26,400	1.6	6.6
PTMSP-1 С2	3000	1700	6400	13,100	1.8	7.7
PTMSP-1 С3	6800	3700	11,800	24,200	1.8	6.5
PTMSP-1 С4	7300	4700	13,200	27,000	1.6	6.7
PTMSP-2	11,500	7800	20,000	38,000	1.5	4.9
PTMSP-2 С1	9400	6300	15,400	35,700	1.5	5.7
PTMSP-2 С2	8100	5300	14,600	29,500	1.5	5.6
PTMSP-2 С3	8000	4700	12,800	26,100	1.7	5.5
PTMSP-2 С4	8200	5300	14,600	29,400	1.6	5.6
PTMSP-3	11,000	7400	19,600	36,900	1.5	5.0
PTMSP-3 С1	11,300	7600	19,000	41,300	1.5	5.4
PTMSP-3 С2	8600	5700	15,200	31,200	1.5	5.5
PTMSP-3 С3	12,500	7400	16,400	33,600	1.7	4.5
PTMSP-3 С4	7800	5300	13,800	28,100	1.5	5.3

* 1 Barrer = 1 × 10^−10^ (cm^3^ (STP) cm cm^−2^ s^−1^ cmHg^−1^).

**Table 3:** The change in permeability coefficients of PTMSP-1 films containing stabilizers Irganox 1010 (С1), Vulkanox BKF (С2), Vulkanox BHT (С3), and Irganox 1076 (C4) during heat treatment (T = 140 °C).

Total Annealing Time, h	Permeability, P (Barrer)				Ideal Selectivity, α	
	O_2_	N_2_	H_2_	CO_2_	O_2_/N_2_	CO_2_/N_2_
PTMSP-1 C1						
48	6800	4500	14,300	27,500	1.5	6.1
72	-	-	-	-	-	-
PTMSP-1 C2						
48	3300	1900	7000	14,000	1.7	7.4
72	-	-	-	-	-	-
PTMSP-1 C3						
48	7400	5900	12,400	21,000	1.8	6.5
72	-	-	-	-	-	-
PTMSP-1 C4						
48	7400	4700	13,200	27,000	1.6	5.7
72	-	-	-	-	-	-

**Table 4:** The change in permeability coefficients of PTMSP-2 films containing stabilizers Irganox 1010 (С1), Vulkanox BKF (С2), Vulkanox BHT (С3), and Irganox 1076 (C4) during heat treatment (T = 140 °C).

Total Annealing Time, h	Permeability, P (Barrer)				Ideal Selectivity, α	
	O_2_	N_2_	H_2_	CO_2_	O_2_/N_2_	CO_2_/N_2_
PTMSP-2 C1						
48	7000	4500	12,700	27,200	1.56	6.04
72	6900	4500	12,300	27,000	1.53	6.00
100	6800	4300	12,200	26,900	1.58	6.26
150	6700	4300	12,100	26,800	1.56	6.23
190	6700	4200	12,000	26,700	1.60	6.36
240	6600	4200	12,000	26,700	1.57	6.36
PTMSP-2 C2						
48	7000	4400	13,300	27,000	1.59	6.14
72	6800	4350	13,100	26,700	1.56	6.14
100	6700	4200	12,900	26,000	1.60	6.19
150	6600	4100	12,700	25,600	1.61	6.24
190	6600	4100	12,600	25,400	1.61	6.20
240	6500	4000	12,500	25,300	1.63	6.33
PTMSP-2 C3						
48	7900	4700	12,500	25,600	1.68	5.45
72	7700	4500	12,800	26,800	1.71	5.96
100	8100	5000	13,200	27,000	1.62	5.4
150	-	-	-	-	-	-
PTMSP-2 C4						
48	6400	4000	12,200	24,700	1.6	6.2
72	6200	3900	12,000	24,400	1.6	6.3
100	6600	3700	11,800	23,800	1.6	6.4
150	6000	3600	11,700	23,600	1.7	6.6
190	5900	3600	11,700	23,500	1.6	6.5
240	5900	3500	11,600	23,300	1.7	6.7

**Table 5:** The change in permeability coefficients of PTMSP-3 films containing stabilizers Irganox 1010 (С1), Vulkanox BKF (С2), Vulkanox BHT (С3), and Irganox 1076 (C4) during heat treatment (T = 140 °C).

Total Annealing Time, h	Permeability, P (Barrer)				Ideal Selectivity, α	
	O_2_	N_2_	H_2_	CO_2_	O_2_/N_2_	CO_2_/N_2_
PTMSP-3 C1						
48	8000	5100	14,100	30,200	1.57	5.92
72	7600	4900	13,600	29,900	1.55	6.10
100	7500	4700	13,200	29,300	1.60	6.23
150	7500	4700	13,000	29,000	1.60	6.17
190	7400	4500	12,800	28,900	1.64	6.42
240	7400	4500	12,700	28,800	1.64	6.40
PTMSP-3 C2						
48	6800	4350	12,800	26,200	1.56	6.02
72	6700	4300	12,600	26,000	1.56	6.05
100	6500	4200	12,400	25,000	1.55	5.95
150	6400	4200	12,200	24,700	1.52	5.88
190	6300	4100	12,100	24,600	1.54	6.00
240	6300	4000	12,000	24,500	1.57	6.13
PTMSP-3 C3						
48	8900	5300	13,500	27,800	1.68	5.25
72	9000	5800	14,700	29,500	1.55	5.09
100	9500	6100	15,000	30,800	1.55	5.04
150	-	-	-	-	-	-
PTMSP-3 C4						
48	5800	3600	11,000	22,400	1.6	6.2
72	5600	3500	10,800	22,000	1.6	6.3
100	5600	3500	10,800	22,200	1.6	6.3
150	5500	3500	10,700	22,000	1.6	6.3
190	5500	3400	10,600	21,800	1.6	6.4
240	5500	3400	10,600	21,700	1.6	6.4

## Data Availability

Not applicable.
